# Bone-Targeted High-Intensity Training Does Not Reduce Quality of Life Related to Pelvic Floor Dysfunction: The MEDEX-OP Randomized Controlled Trial

**DOI:** 10.1007/s00192-025-06187-x

**Published:** 2025-06-16

**Authors:** Melanie Kistler-Fischbacher, Benjamin K. Weeks, Belinda R. Beck

**Affiliations:** 1 School of Health Sciences and Social Work, Griffith University, Gold Coast Campus, Gold Coast, QLD 4222 Australia; 2 Department of Aging Medicine and Aging Research, University of Zurich, Zurich, Switzerland; 3 The Bone Clinic, Brisbane, Australia

**Keywords:** Bone health, Weightlifting, Women’s health

## Abstract

**Introduction and Hypothesis:**

High-intensity resistance and impact training (HiRIT) is the most effective exercise for improving bone strength, but it is traditionally discouraged for individuals with pelvic floor dysfunction, a condition that often coexists with osteoporosis. We therefore aimed to compare the effect of HiRIT with low-intensity Pilates-based exercise (LiPBE) on quality of life related to pelvic floor dysfunction in postmenopausal women.

**Methods:**

Healthy postmenopausal women with low bone mass were recruited and randomized to 8-month, twice-weekly supervised HiRIT or LiPBE. Pelvic floor-related quality of life was assessed using the Pelvic Floor Disability Index (PFDI-20) and Pelvic Floor Impact Questionnaire (PFIQ-7). Subgroup analysis was conducted to explore the overall effect of exercise (HiRIT or LiPBE) on PFDI-20 and PFIQ-7 total scores in individuals with and without a history of pelvic organ prolapse (POP).

**Results:**

A total of 115 women were included (mean age 64.4 years). There were no significant between-group differences in 8-month change in the PFDI-20 or its subscales, or the PFIQ-7. There was a significant within-group improvement in the Pelvic Organ Prolapse Distress Inventory subscale of the PFDI-20 (−2.3 [95% CI −4.4, 0.2]) in the LiPBE group. Exploratory subgroup analyses of the combined exercise groups showed an improvement in the PFDI-20 among participants with a history of POP over the 8-month training period (−11.9 [95% CI −22.8, −1.1]).

**Conclusion:**

Our findings suggest no negative effects of bone-targeted HiRIT on pelvic floor-related quality of life in postmenopausal women. On the contrary, our data provide preliminary evidence that exercise may have beneficial effects on pelvic floor health. However, further studies are needed.

## Introduction

Osteoporosis or low bone mass, and pelvic floor dysfunction are prevalent comorbidities in postmenopausal women that greatly impact quality of life [1, 2]. In Australia, approximately one in two women over the age of 50 years has low bone mass or osteoporosis [3] and 43% of those over 65 years have at least one pelvic floor disorder [4].

Pelvic floor dysfunction includes disorders related to pelvic organ prolapse (POP), urinary or fecal incontinence and pelvic pain [5]. Although these disorders rarely result in severe morbidity, they can negatively impact mental health (e.g., stress, anxiety), physical performance, sexual function, and social participation, and thereby affect quality of life [2, 6–8].

Exercise is an important strategy in the management of both conditions; however, prescriptions differ markedly. Bone-targeted exercise to reduce fracture risk in postmenopausal women should include high-intensity resistance and impact training (HiRIT) [9]. Specifically, resistance training at loads corresponding to > 80% of the one repetition maximum (1RM) and impact training generating ground reaction forces greater than four times bodyweight are required to stimulate an adaptive bone response [10]. In contrast, recent guidelines from the National Institute for Health and Care Excellence on the nonsurgical management of pelvic floor dysfunction discourage heavy lifting [11]. The recommendation is based on the assumption that the increase in intra-abdominal pressure when lifting weight will further damage the pelvic floor. There are no high-quality clinical trial data underpinning this conservative recommendation [11].

The present short report describes secondary pelvic floor outcomes from the Medication and Exercise for Osteoporosis (MEDEX-OP) trial. Our aim was to examine the effect of HiRIT in comparison with low-intensity Pilates-based exercise (LiPBE) on quality of life related to pelvic floor dysfunction in postmenopausal women with low bone mass or osteoporosis. We hypothesized that there would be no difference between the two exercise interventions in this outcome measure.

## Materials and Methods

### Study Design and Participants

The MEDEX-OP trial was an 8-month, participant-blinded, two-arm, randomized controlled trial. The detailed protocol [12] and primary and secondary outcomes related to bone health and fracture risk [13, 14] have been published previously. In brief, we recruited women who were at least 5 years post menopause and had low bone mass or osteoporosis (lumbar spine and/or femoral neck T score ≤ −1.0). Participants had to be free of medical conditions and medications known to influence bone health (e.g., glucocorticoids, diabetes), recent fractures and injuries or medical conditions that could prevent completion of the exercise programs. Furthermore, participants had to be willing and able to attend twice-weekly exercise classes for 8 months.

Eligible participants were block randomized (block size of four) with a 1:1 allocation ratio to HiRIT or LiPBE. The allocation sequence was computer generated and sequentially sealed in opaque envelopes. Allocation was concealed from the tester and participants until completion of the baseline assessment. Owing to the nature of exercise interventions, participants could not be blinded to group allocation; however, blinding to the study hypotheses prevented them from knowing which exercise program was expected to be more beneficial.

### Exercise Interventions: High-Intensity Resistance and Impact Training

Both exercise interventions included twice-weekly, 40-min supervised sessions on nonconsecutive days for 8 months (35 weeks, 70 sessions). All sessions were supervised by an Exercise Scientist or Physiotherapist. Attendance was recorded and adherence calculated as a percentage of the maximum potential sessions attended.

The HiRIT (Onero™) included three resistance training exercises (deadlift, back squat, overhead press), conducted in five sets of five repetitions at > 80% of the 1RM. A 6 to 20 Borg scale was used to monitor training intensity with the aim of achieving a rating of ≥ 16 (“very hard”) for exercise in each session. The impact exercise was an assisted jump with stiff-legged landing, using the same scheme of five sets of five repetitions. Exercises were introduced gradually over a 2-week accommodation period, with a focus on technique and safe postures. Each session also included two balance exercises.

The LiPBE (Buff Bones®) followed the Buff Bones® movement system and focused on whole-body strength, balance, mobility, and posture. Exercises were mainly Pilates-based and performed on the mat in supine, prone, quadruped, and side-lying positions (e.g., single-leg stretch, clam shells, single-leg kick), as well as a number of standing, weight-bearing exercises including impact (e.g., stomping), light dumbbell repetitions (e.g., triceps extension, bent-over row), and squats. A detailed description can be found elsewhere [12].

### Outcome Measures

Pelvic floor-related quality of life was included as a secondary outcome in the MEDEX-OP trial owing to the high prevalence of pelvic floor disorders in women with osteoporosis. The Pelvic Floor Disability Index (PFDI-20) and Pelvic Floor Impact Questionnaire (PFIQ-7) were chosen because they are validated, widely used, and simple to administer. Both questionnaires were developed and validated in English to assess pelvic floor-specific quality of life [8, 15]. The PFDI-20 short form contains 20 items across three scales: the Urinary Distress Inventory (UDI), the Pelvic Organ Prolapse Distress Inventory (POPDI), and the Colorectal-Anal Distress Inventory (CRADI). The PFIQ-7 short form includes seven items, which are rated across three domains: bladder/urine, bowel/rectum, and vagina/pelvis. All scales in these short forms show a significant correlation with their respective long-form versions [15]. Both questionnaires were self-administered and completed at baseline and after the 8-month exercise interventions. Scores on both questionnaires range from 0 to 300, with higher scores reflecting a more negative impact of pelvic floor symptoms on quality of life [15]. For the PFDI-20, the POPDI-6, CRADI-8, and UDI-6 subscales were calculated and analyzed in addition to the total score. Evaluation of subscales for the PFIQ-7 was not possible, as the total scores of the PFIQ-7 were low and many of the subscale scores were 0.

### Statistical Analysis

Baseline characteristics of participants were described overall and by intervention group and compared using *t* tests for normally distributed continuous variables, Mann–Whitney *U* test for non-normally distributed continuous data, and Chi-squared test for categorical data. Between-group differences of 8-month change in outcome measures were evaluated using repeated measure analysis of variance (RMANOVA). Two separate models were run. The first was adjusted for initial values only and the second one was adjusted for initial values and compliance, as specified in the protocol [12]. The reason for running both models was the reduced sample sizes for results adjusted for compliance. Compliance was based on the percentage of classes attended during the intervention period, in relation to the total possible sessions with 100% compliance (e.g., for 35 weeks, 70 sessions equals 100% compliance) [16]. We then adjusted the statistical comparisons using percentage compliance. Intention-to-treat analyses were adopted (including all participants randomized at baseline). Missing values were imputed using the mean absolute change value of the respective group.

In an exploratory subgroup analysis, we examined the overall effect of exercise (combining HiRIT and LiPBE) on PFDI-20 and PFIQ-7 total scores in individuals with a history of POP and those without. We justify the approach of combining the two exercise groups with the limited existing evidence on the effects of general exercise on pelvic floor health as well as the small sample size of women with POP in the present trial.

The SPSS Statistics software, version 29.0 (IBM, Armonk, NY, USA) was used and all analyses were examined against a significance of *p* ≤ 0.05.

## Results

### Participant Characteristics

From a total of 880 volunteers, 115 women met the eligibility criteria and were included in the MEDEX-OP trial and randomized to HiRIT (*n* = 57) or LiPBE (*n* = 58). Eleven participants withdrew from the trial so that 104 (93.3%) completed their 8-month intervention. The CONSORT diagram of participant flow has been published [13].

Participant characteristics at baseline are presented in Table 1. Overall, the mean age was 64.4 (SD 6.1) years, participants were on average 15.0 (7.6) years post menopause, and the mean femoral neck T score was −1.9 (0.8). Ninety-one (79.1%) participants reported a history of vaginal delivery, 15 (13.0%) POP, and 23 (20.0%) a hysterectomy. There were no significant differences in those characteristics between the HiRIT and LiPBE groups at baseline (Table 1).

**Table 1:** Baseline characteristics of study participants

Characteristic	Overall (*n* = 115)	LiPBE (*n* = 58)	HiRIT (*n* = 57)	*p* value
Age (years), mean (SD)	64.4 (6.2)	64.2 (5.5)	64.6 (6.8)	0.92
Years post menopause, mean (SD)	15.0 (7.6)	14.9 (8.2)	15.0 (7.0)	0.63
BMI (kg/m^2^), mean (SD)	25.5 (4.4)	25.4 (4.5)	25.6 (4.4)	0.56
Lumbar spine T score, mean (SD)	−1.8 (1.1)	−1.6 (1.1)	−2.0 (1.0)	0.07
Femoral neck T score, mean (SD)	−1.9 (0.8)	−2.0 (0.6)	−1.9 (0.9)	0.40
History of vaginal delivery, *n* (%)	91 (79.1)	48 (83%)	43 (76%)	0.33
Pelvic organ prolapse, *n* (%)	15 (13.0)	7 (12%)	8 (14%)	0.75
Hysterectomy, *n* (%)	23 (20.0)	9 (16%)	14 (25%)	0.23
PFDI-20 total score, mean (SD)	32.6 (35.1)	34.6 ± 35.9	30.5 ± 34.5	0.28
PFIQ-7 total score, mean (SD)	4.1 (11.7)	3.9 ± 11.1	4.3 ± 12.4	0.92

*BMI* body mass index, *HiRIT* high-intensity resistance and impact training, *LiPBE* low-intensity Pilates-based exercise, *PFDI-20* Pelvic Floor Disability Index 20, *PFIQ-7* Pelvic Floor Impact Questionnaire 7

Mean adherence with the exercise programs was 83.1 (SD 12.1) % for HiRIT and 81.6 (13.7) % for LiPBE.

### Intervention Effects on Pelvic Floor Disability Index and Pelvic Floor Impact Questionnaire

For PFDI-20 and PFIQ-7, negative changes in scores reflect improvement whereas positive changes reflect worsening of pelvic floor-related quality of life. There were no significant differences in 8-month change in PFDI-20 total score or its subscales between the LiPBE and HiRIT groups, but there was a within-group decrease in the POPDI-6 subscale (Pelvic Organ Prolapse Distress Inventory) in the LiPBE group in the unadjusted analysis (−2.3, 95% CI −4.4, −0.2; Tables 2, 3) not evident in the analysis adjusted for compliance. Similarly, there was no significant between-group difference in 8-month change in the PFIQ-7. Results adjusted for exercise compliance align with these findings (Table 3).

**Table 2:** Baseline, 8-month, and mean change unadjusted values (95% CI) for the Pelvic Floor Disability Index 20 (PFDI-20) and the Pelvic Floor Impact Questionnaire 7 (PFIQ-7)

Outcome, unadjusted	LiPBE (*n* = 58)			HiRIT (*n* = 57)			*p* value
	Baseline	Follow-up	Change	Baseline	Follow-up	Change	
PFDI-20 total	34.6 (25.5, 43.8)	29.8 (21.7, 37.9)	−4.8 (−10.4, 0.8)	30.5 (21.2, 39.7)	26.1 (17.9, 34.2)	−4.4 (−10.0, 1.2)	0.92
POPDI-6	8.8 (5.7, 11.8)	6.5 (3.7, 9.3)	−2.3 (−4.4, −0.2)*	7.2 (4.1, 10.3)	6.9 (4.1, 9.8)	−0.3 (−2.4, 1.8)	0.19
CRADI-8	12.1 (8.7, 15.4)	11.2 (8.2, 14.2)	−0.9 (−2.9, 1.2)	10.3 (6.9, 13.7)	8.4 (5.4, 11.5)	−1.9 (−3.9, 0.1)	0.47
UDI-6	13.8 (9.8, 17.8)	12.1 (8.5, 15.8)	−1.7 (−4.7, 1.4)	12.9 (8.9, 17.0)	10.7 (7.0, 14.4)	−2.2 (−5.3, 0.8)	0.80
PFIQ-7	3.9 (0.8, 7.0)	1.8 (−0.9, 4.4)	−2.1 (−4.3, 0.1)	4.3 (1.2, 7.3)	4.1 (1.4, 6.7)	−0.2 (−2.4, 2.1)	0.27

*CRADI-8* Colorectal-Anal Distress Inventory 8, *HiRIT* high-intensity resistance and impact training, *LiPBE* low-intensity Pilates-based exercise, *POPDI-6* Pelvic Organ Prolapse Distress Inventory 6, *UDI-6* Urinary Distress Inventory 6

*Within-group change from baseline *p* < 0.05

**Table 3:** Baseline, 8-month, and mean change adjusted values (95% CI) for the Pelvic Floor Disability Index 20 (PFDI-20) and the Pelvic Floor Impact Questionnaire 7 (PFIQ-7)

Outcome, adjusted	LiPBE (*n* = 52)			HiRIT (*n* = 52)			*p* value
	Baseline	Follow-up	Change	Baseline	Follow-up	Change	
PFDI-20 total	33.3 (23.6, 42.9)	28.8 (20.4, 37.2)	−4.5 (−10.7, 1.8)	33.6 (24.0, 43.2)	28.9 (20.6, 37.2)	−4.7 (−10.8, 1.5)	0.96
POPDI-6	8.5 (5.2, 11.8)	6.3 (3.3, 9.3)	−2.2 (−4.5, 0.1)	8.4 (5.1, 11.6)	7.9 (4.9, 10.8)	−0.5 (−2.8, 1.8)	0.31
CRADI-8	11.7 (8.1, 15.3)	10.9 (7.7, 14.1)	−0.8 (−3.1, 1.4)	10.7 (7.2, 14.3)	8.9 (5.8, 12.1)	−1.8 (−4.0, 0.4)	0.53
UDI-6	13.1 (8.9, 17.2)	11.6 (7.9, 15.3)	−1.5 (−4.9, 1.9)	14.5 (10.4, 18.6)	12.1 (8.5, 15.8)	−2.4 (−5.7, 1.0)	0.71
PFIQ-7	4.0 (0.6, 7.3)	1.9 (−1.0, 4.7)	−2.1 (−4.6, 0.4)	4.5 (1.2, 7.8)	4.3 (1.4, 7.1)	−0.2 (−2.7, 2.2)	0.28

*CRADI-8* Colorectal-Anal Distress Inventory 8, *HiRIT* high-intensity resistance and impact training, *LiPBE* low-intensity Pilates-based exercise, *POPDI-6* Pelvic Organ Prolapse Distress Inventory 6, *UDI-6* Urinary Distress Inventory 6

### Results From Exploratory Subgroup Analyses

Although there was an evident trend in the combined exercise group analysis toward a greater reduction in PFDI-20 score in individuals with a history of POP than in those without POP (−11.9 [95% CI −22.8, −1.1] vs −3.5 [95% CI −7.7, 0.7]), it was not statistically significant (*p* = 0.15). This trend toward improvement in the group with a history of POP was reflected in a statistically significant within-group reduction in PFDI-20 score (*p* = 0.03).

There were no significant between-group differences in 8-month change in PFIQ-7 scores, with a history of POP and those without POP (2.2 [95% CI −2.1, 6.6] vs −1.7 [95% CI −3.4, 0.01], *p* = 0.10) and no within-group differences.

## Discussion

The present study examined the effects of HiRIT compared with LiPBE on quality of life related to pelvic floor dysfunction. There were no significant differences in 8-month change in overall pelvic floor-related quality of life within or between the two treatment groups. This study confirms our hypothesis that HiRIT does not aggravate symptoms of pelvic floor dysfunction, and the effect does not differ from that of a LiPBE intervention.

The present study adds novel data and insight into the effect of exercise on pelvic floor health in postmenopausal women. To the best of our knowledge, it is the first randomized controlled trial to examine the effect of two different exercise programs, which did not include specific pelvic floor muscle training (PFMT), on pelvic floor health in postmenopausal women. Our findings align with those of a 12-week RCT that compared the effect of PFMT in combination with weight training at loads corresponding to 15 repetitions maximum (approximately 65% 1RM [17]) and PFMT alone in 32 women (> 60 years of age) with urinary incontinence [18]. After 4 weeks, the combined weight training plus PFMT group achieved a greater rate of absence of symptoms (58.3%) than the PFMT alone group (14.3%). At the end of the 12-week intervention period, this proportion increased to 75.0% in the combined group and 35.7% in the PFMT alone group. The authors suggest that the weight training provided additional stimuli to the pelvic floor and thus reduced symptoms faster and more successfully than PFMT alone [18].

Data from observational studies also support our findings. In the Nurses Health Study, women (age range 37–54 years) in the highest quintile of recreational physical activity participation had a 20% lower risk of urinary incontinence, compared with the lowest quintile. Furthermore, higher levels of physical activity were associated with a 25–30% lower risk of developing stress incontinence, defined as leakage occurring with coughing, sneezing, lifting, laughing, and exercise [19]. With respect to lifting weights, a cross-sectional study of 3934 individuals (mean age 40.3 years) examined the association of no exercise, lifting heavy (≥ 50 kg), moderate (16–50 kg), or light (≤ 15 kg) weights and self-reported symptoms of POP [20]. The proportion of participants reporting symptoms was significantly lower in the heavy-lifting group (7.1%) than in the light-lifting (19.4%) and inactive (21.3%) groups. Another cross-sectional study reported significantly lower symptoms of POP and anal incontinence in women who engage in CrossFit®, a training modality including heavy lifting, high-impact exercises, and functional movements, compared with recreational runners (POP 7.8% vs 12.7%; anal incontinence 27.7% vs 34.0%, *p* < 0.05) [21].

We performed exploratory subgroup analyses, combining the two exercise groups to improve power, to explore the general effect of exercise among participants with a history of POP. Results show an improvement in quality of life related to pelvic floor dysfunction specifically among women with a history of POP. Although exploratory, these findings are of clinical relevance and align with the evidence summarized above, that bone-targeted therapeutic exercise, including high-intensity resistance training and high-impact exercise, does not negatively affect pelvic floor health and may even have a protective effect.

In contrast to the positive effects of exercise on pelvic floor health summarized above, we also acknowledge the body of literature that reports inconclusive or harmful effects of exercise in general and weight lifting in particular [22–24]. A recent review summarizes in detail the evidence for the positive effects as well as harmful effects of exercise on pelvic floor health [24]. More importantly, the review identified several knowledge gaps that prohibited firm conclusions on the impact of high-intensity activities on pelvic floor health, suggesting that further high-quality studies might be needed.

This study has some limitations. First, the PFDI-20 and PFIQ-7 measure the impact of pelvic floor dysfunction on quality of life and do not provide a measure of symptom severity. However, they correlate well with appropriate measures of symptom severity and pelvic floor diagnoses [15]. Second, participants in this study were not specifically selected for pelvic floor dysfunction so our findings may not translate broadly to that population. Third, both groups engaged in a supervised, twice-weekly exercise program, which prohibited comparison with a non-exercise control group.

## Conclusion

Our findings suggest no negative effects of bone-targeted HiRIT on pelvic floor-related quality of life in postmenopausal women. On the contrary, our data provide preliminary evidence that high intensity resistance and impact training may have a protective effect among women with a history of POP. Further studies examining the effect of heavy lifting on symptom severity are needed, particularly in individuals with specific pelvic floor disorders.

## Data Availability

The data that support the findings of this study are available on reasonable request from the corresponding author. The data are not publicly available owing to privacy or ethical restrictions.
